# Nucleoid-Associated Proteins Affect Mutation Dynamics in *E. coli* in a Growth Phase-Specific Manner

**DOI:** 10.1371/journal.pcbi.1002846

**Published:** 2012-12-20

**Authors:** Tobias Warnecke, Fran Supek, Ben Lehner

**Affiliations:** 1 Bioinformatics and Genomics Program, Centre for Genomic Regulation (CRG), Barcelona, Spain; 2 Universitat Pompeu Fabra (UPF), Barcelona, Spain; 3 EMBL-CRG Systems Biology Unit, Centre for Genomic Regulation (CRG), Barcelona, Spain; 4 Institució Catalana de Recerca i Estudis Avançats, Centre for Genomic Regulation (CRG) and UPF, Barcelona, Spain; CNAG - Centre Nacional d'Anàlisi Genòmica and CRG - Centre de Regulació Genòmica, Spain

## Abstract

The binding of proteins can shield DNA from mutagenic processes but also interfere with efficient repair. How the presence of DNA-binding proteins shapes intra-genomic differences in mutability and, ultimately, sequence variation in natural populations, however, remains poorly understood. In this study, we examine sequence evolution in *Escherichia coli* in relation to the binding of four abundant nucleoid-associated proteins: Fis, H-NS, IhfA, and IhfB. We find that, for a subset of mutations, protein occupancy is associated with both increased and decreased mutability in the underlying sequence depending on when the protein is bound during the bacterial growth cycle. On average, protein-bound DNA exhibits reduced mutability compared to protein-free DNA. However, this net protective effect is weak and can be abolished or even reversed during stages of colony growth where binding coincides – and hence likely interferes with – DNA repair activity. We suggest that the four nucleoid-associated proteins analyzed here have played a minor but significant role in patterning extant sequence variation in *E. coli*.

## Author Summary

Mutations can be more or less likely to occur depending on whether DNA is naked or bound by proteins. On the one hand, DNA-binding proteins can shield the DNA from certain mutagenic processes. On the other hand, the very same proteins can interfere with efficient DNA repair. In this study, we reconstruct the history of mutations across 54 *E. coli* genomes and ask whether mutation risk is higher or lower in regions occupied by proteins that help organize bacterial DNA into chromatin. Intriguingly, we find that the effect of binding depends on its timing. When we consider genomic regions bound during stationary phase, we observe that binding is associated with lower mutation risk for some mutation classes compared to naked DNA, albeit weakly. However, when binding occurs during exponential phase, bound regions actually experience more mutations on average. We argue that this is because, during exponential phase, the major effect of binding is that it interferes with efficient DNA repair, whereas in stationary phase – when many repair pathways are inactive – the protective effect of binding dominates. Our results suggest that the four DNA-binding proteins considered here have a small but significant growth phase-specific effect on mutation dynamics in *E. coli*.

## Introduction

The binding of proteins to DNA can alter DNA mutability. This has been explored most extensively in relation to nucleosomes. On the one hand, there is evidence that nucleosomes affect the ability of at least some repair enzymes to detect or gain access to their target lesions [1], [2]. Typically, nucleosomes appear to impede rather than facilitate repair, for example through steric hindrance or by altering DNA topology, which many repair enzymes exploit to recognize their targets [2]–[4]. Human uracil glycosylases, for instance, operate with 3- to 9-fold reduced efficiency *in vitro* when uracil needs to be removed in a nucleosome context compared to free DNA [5]. On the other hand, histone binding can also lower mutability by reducing the risk of lesion formation. In yeast, nucleosomal DNA exhibits reduced rates of cytosine deamination [6], probably because the nucleosome conformation reduces the amount of time the DNA spontaneously spends in a single-stranded state [6], which is associated with an elevated risk of deamination [7]. Being in close contact with a protein surface can also more directly inhibit mutagenic processes, as illustrated at the sub-nucleosome scale by the observation that residues facing the histone core have a lower propensity to form pyrimidine dimers [8]. Nucleosomes, therefore, can exert pleiotropic and contravening effects on sequence mutability, both interfering with repair and conferring physical protection.

Although bacteria do not encode histones they do express a variety of DNA-binding proteins. Some, collectively known as nucleoid-associated proteins (NAPs), are abundant in the cell and fulfil both architectural and regulatory roles [9]. For a subset of NAPs, there is evidence from mutation-specific reporter strains that their presence too can affect sequence mutability. Notably, the DNA-protection during starvation (Dps) protein, expressed at high levels in stationary phase [10] and during oxidative stress [11] in *E. coli*, reduces the incidence of double strand breaks as well as C∶G to A∶T transversions [11], [12]. Similarly, small acid soluble proteins (SASPs), present in the spores of *Bacillus subtilis*, limit the formation of certain lesions including pyrimidine dimers [13]. Part of the protective effect may be indirect and global. Dps, for example, sequesters iron and thereby reduces the formation of mutagenic agents [14]. But there is also evidence for localized protection, where protein binding alters DNA mutability in and around the binding footprint, through direct physical interaction or by virtue of modulating repair dynamics [15], [16].

As NAPs constitute a diverse class of proteins, variously able to wrap, bridge, or bend DNA, it may come as no surprise that not all NAPs necessarily reduce lesion formation. The abundant DNA-binding protein Fis, for example, appears to promote the induction of (specifically) pyrimidine dimers [16], likely because its binding locally alters DNA curvature, making it more conducive to dimer formation [17]. In short, biochemical studies provide strong evidence that a number of different NAPs can affect DNA mutability in a number of different ways, variously able to confer localized protection, remodel DNA topology and thereby render DNA more prone to mutations, or – like Dps – modulate mutation risk systemically.

Despite strong *in vitro* (and short-term lab culture) evidence from both eukaryotic and prokaryotic systems that protein occupancy can affect mutation dynamics, our knowledge of how repair and protective effects trade off to influence mutability *in vivo* and, ultimately, pattern segregating and fixed variation in natural populations remains rudimentary. Several genome-scale studies of polymorphism and divergence patterns in eukaryotes have found rates of evolution to vary with nucleosome occupancy [18]–[24] and a recent analysis of multiple cancer genomes uncovered striking correlations between different chromatin modifications and regional mutation rates [25]. However, it remains largely unclear whether correlations with nucleosome occupancy and chromatin status reflect differential repair efficacy, mutational liability or – especially where inter-specific comparisons are concerned – selection, for example for adequate nucleosome positioning. In fact, when it comes to repair, it is difficult to say whether we should have strong *a priori* expectations. Despite the stark differences in repair efficacy observed *in vitro*, histones and other DNA-binding proteins might be rather permeable barriers to repair *in vivo* where chromatin remodelers and polymerases regularly evict these proteins, provide opportunities for repair [26] and therefore potentially negate occupancy effects [27].

One approach to revealing how repair and protection affect heterogeneous mutability across the genome is to contrast the evolution of neutrally evolving sequences (thus by-passing selection as a potential confounder) bound under different physiological conditions (e.g. exponential versus stationary phase growth). The idea here is simple: being refractory to repair only matters if the relevant repair machinery is actually active at the time when the focal protein is bound. Conversely, protection will be of greater importance whenever the risk of mutagenesis is higher. Thus, in principle, analyzing physiologically specific binding events might allow us to look beyond the net impact on mutability and reveal insights into the relative sway of protective and repair effects. In practice, nucleosome binding profiles – at least in budding and fission yeast - change little when entering stationary phase, during heat shock or even in cells undergoing meiosis [28]–[30]. The binding landscapes of some bacterial NAPs, however, change much more radically throughout growth [9], [31], [32]. In this study, we therefore analyze data from a series of Chip-Seq experiments in *E. coli* where binding profiles of four NAPs (H-NS, Fis, IhfA and IhfB) were determined on a genome-wide scale [31], [32]. Relating NAP binding profiles assayed during different stages of the *E. coli* growth cycle to patterns of sequence evolution across 54 *E. coli* strains, we present evidence for weak but significant growth stage-specific effects of protein occupancy on mutability that reveal a dynamic balance between protective and repair effects.

## Results

In order to establish whether the binding of different NAPs affects mutability we first reconstructed the history of single nucleotide changes across 54 *E. coli* strains (see Methods). As the strains are closely related, observed changes represent relatively recent events, and, at least for a subset of sites (see below), likely reflect mutational input rather than selection [33].

For a first coarse-grained view, we classified genomic regions into those bound by one of the NAPs at any point during colony growth versus regions that are never bound by any of the NAPs considered. For all possible nucleotide replacements, we then considered mutability as a function of NAP binding assayed in the K-12 MG1655 strain [31], [32]. Mutability (or mutation risk) is defined here as the number of changes observed across the phylogeny divided by the number of at-risk nucleotides. For example, we would count all C to T changes that occurred at intergenic sites located in Fis-bound DNA and then divide this tally by the number of all cytosine located in intergenic Fis-bound DNA, i.e. by all sites that fall into the same binding/site category (Fis-bound, intergenic) and could, in principle, have experienced the same mutation.

**Figure 1:**
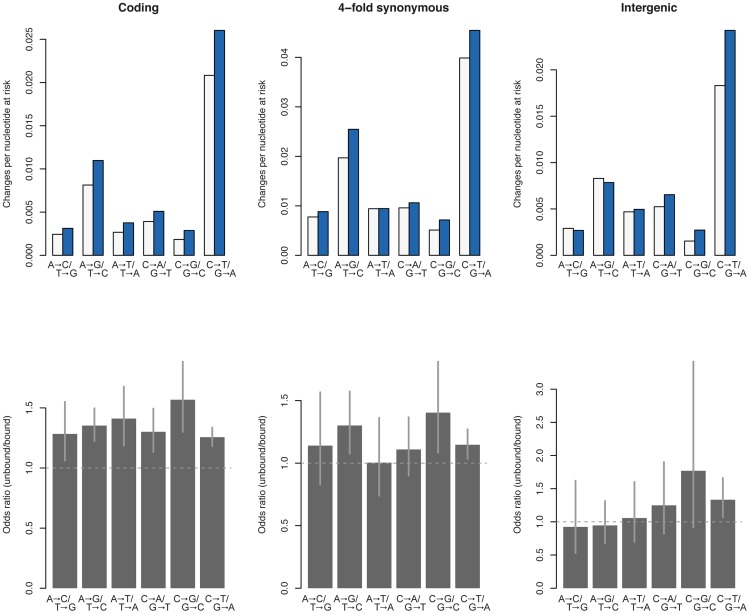
Mutability as a function of NAP occupancy. The upper panels depict mutability (changes per nucleotide at risk) as a function of NAP occupancy for all possible transitions and transversions. White: sequence bound by one of the four NAPs. Sequence is considered bound when at least one of the NAPs binds during at least one of the growth phases assayed (see main text). Blue: sequence not bound by any of the four NAPs throughout growth. The lower panels show odds ratios along with 95% confidence intervals. Values in excess of 1 indicate higher mutability in unbound sequence.

Figure 1 suggests that there is a tendency for unbound sequence to have higher mutability than NAP-bound sequence. However, estimates are not entirely consistent between coding, intergenic, and 4-fold synonymous sites (where all three possible nucleotide replacements do not result in a different amino acid) suggesting that selection confounds our estimates of NAP binding effects. Indeed, as one might expect, the different site classes considered here are subjects to different evolutionary regimes. Notably, the average probability for a nucleotide change to occur is ∼2-fold lower in coding sequence as a whole compared to 4-fold synonymous sites, indicating that – in the former case – we are looking at the combined effect of mutation and selection. Intergenic sites show similarly reduced rates of change. The intergenic regions considered here likely harbour conserved regulatory elements because, when reconstructing sequence evolution, we required homologous regions to be present across all 54 genomes (as well as *E. fergusonii*, see Methods). This strongly implies that analyzing either nonsynonymous or intergenic nucleotides will lead to a misleading view of NAP-related mutability. For the remainder of the analysis we therefore focus on 4-fold synonymous sites, where selection is much reduced or entirely absent [33] and which should therefore reproduce most faithfully the underlying mutation dynamics. In fact, reassuringly, the spectrum of nucleotide changes at 4-fold synonymous sites observed here resembles the frequency spectrum obtained in a recent mutation accumulation experiment [34], with transitions outnumbering transversions and C∶G to T∶A transitions by some distance the most common type of mutation (Figure S1).

### NAPs affect mutability in a growth phase-specific manner

**Figure 2:**
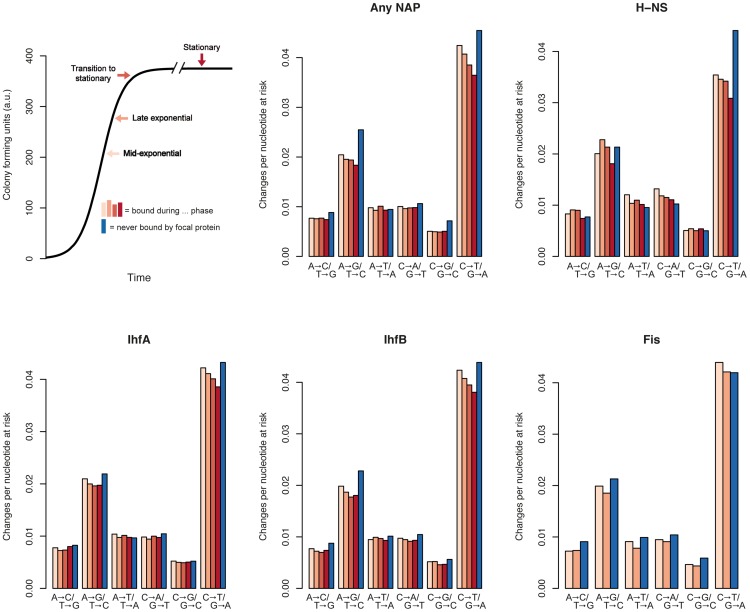
Growth phase dependency of mutability in NAP-bound sequence. The top left panel shows schematically when NAP binding was assayed by Chip-Seq during the *E. coli* growth cycle. The remaining panels depict mutability at 4-fold synonymous sites as a function of the binding of a specific NAP (or of all NAPs combined) at a specific stage during the growth cycle. The colour coding corresponds to the colours of the growth phase labels in the top left panel. Note that mutability estimates for a certain growth phase do not only include regions that are exclusively bound during that phase but also include regions that are bound at several stages during the growth cycle.

Next, we assessed mutability for sequence bound by a specific NAP at a defined stage of the growth cycle (during mid-exponential, late exponential, transition to stationary, and/or stationary phase). The most striking pattern to emerge is that, for C∶G to T∶A transitions in particular, mutability appears to change systematically with the time of binding (Figure 2). This is the case for all NAPs considered (note that Fis is only found at detectable levels during mid- and late exponential phase). Overall, regions bound later during the growth cycle seem to experience successively reduced mutability.

Although these trends are intriguing, systematic biases may suggest a causal growth phase-specific association between protein occupancy and mutability when, in fact, there is none. For example, binding regions might differ by growth stage with regard to their expression level and hence opportunities to benefit from transcription-coupled repair. To establish whether there is an independent effect of binding status on mutability we trained a series of Random Forest classifiers to discriminate between at-risk 4-fold synonymous sites that had experienced a change and those that had not. Along with growth phase-specific NAP binding status, the classifiers were provided with features such as expression, genomic location, and regional GC content (see Methods for a full list of features) that might alternatively account for a difference in mutability between bound and unbound sequence. Note that some of these features relate to strand-specific processes (replication and transcription); we therefore trained a total of 12 (rather than six) classifiers, considering, for example, C to T changes on the transcribed strand as distinct from C to T changes on the non-transcribed strand. For each classifier, we then asked whether the predictive power of the classifier dropped when NAP binding status was randomized across sites, repeating the randomization plus classification procedure 50 times to establish significance. A significant drop in classifier performance means that NAP binding contains information relevant for predicting mutability that cannot be reproduced from any of the other variables, either alone or in combination.

Focusing on those mutation categories where Figure 1 suggested a possible role for NAP binding, we find that, upon randomizing NAP occupancy, classifier performance drops significantly for C to G, G to C, C to T and T to C changes (Figure S2). Interestingly, NAP binding is not predictive of either A to G or G to A mutability, suggesting strand-specific effects. Overall, our results suggest that NAP binding is a weak predictor of net mutation risk, particularly when compared to other features in the analysis. For example, location on leading versus lagging strand has a considerably stronger influence on mutability (not shown), consistent with results from a recent mutation accumulation experiment [34]. However, as we argue below, this weak net effect of NAP binding conceals a more dynamic picture of the interplay between *E. coli* chromatin and mutability.

### Protective effects alone cannot explain patterns of growth phase-dependent mutability

Having established that binding is an independent, albeit weak predictor of mutability at 4-fold synonymous sites for a subset of mutation types, we wondered why mutability should be relatively higher for DNA bound during exponential growth. There are a number of scenarios that might explain this pattern: one simple explanation might be that there is an interaction between a protective effect of NAP binding and the timing of mutations. Imagine all mutations occurred during stationary phase so that the protective effect would be mediated exclusively by stationary phase binding. In this scenario, regions bound during exponential phase should behave the same as unbound sequence. However, we may still observe a signature of protective binding if early-bound regions are also bound in stationary phase. Such overlap in binding sites is indeed observed, with temporally more remote growth phases sharing increasingly fewer binding sites (Figure S3). A second possibility is that binding is more dynamic during exponential phase where protein occupancy is constantly disturbed by polymerases so that earlier growth phases contain a greater fraction of bound sites that spend a considerable amount of time in an unbound state, and therefore only benefit partially from the protective effect of binding.

**Figure 3:**
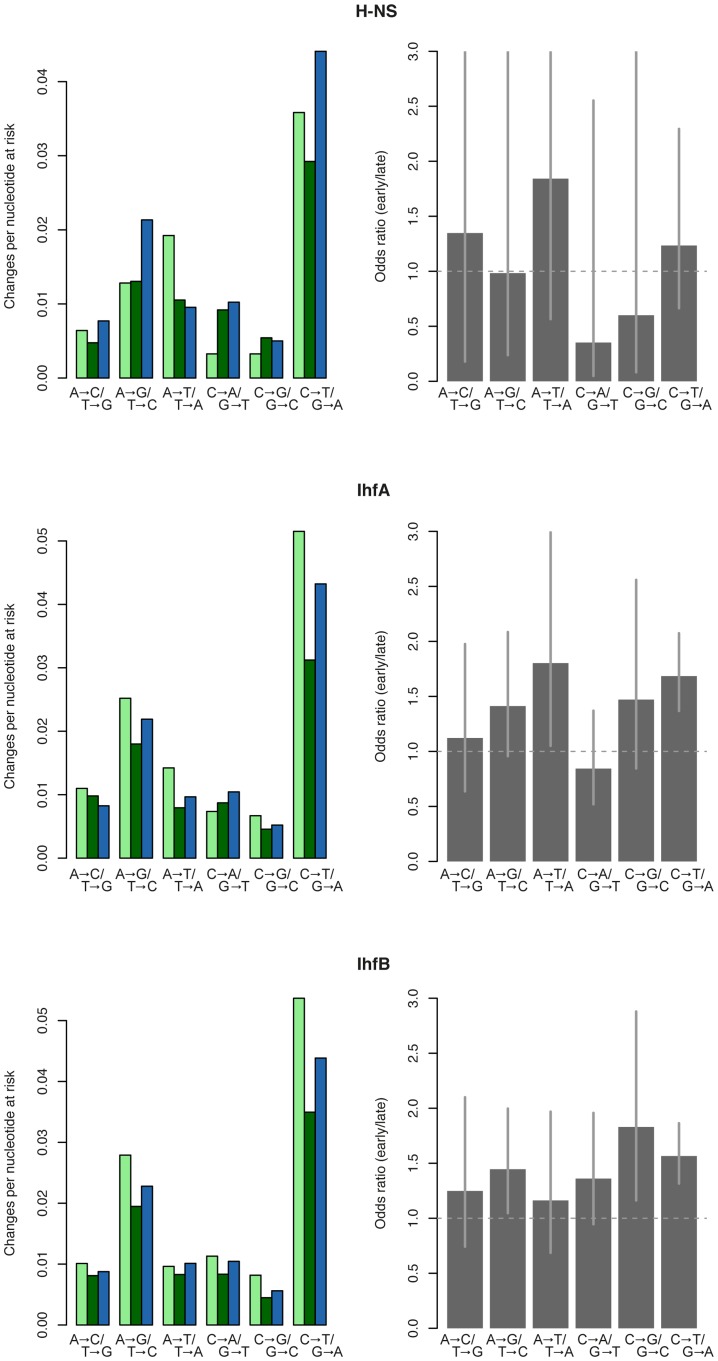
Growth phase-specifically bound sequence reveals time-dependent effects of NAP binding on mutability. Left-hand panels: Mutability as a function of H-NS, IhfA, or IhfB occupancy for all possible transitions and transversions. Light green: sequence bound by the focal protein during mid- and/or late exponential phase but not later. Dark green: sequence bound by the focal protein during transition to stationary and/or stationary phase but not earlier. Blue: sequence not bound by the focal NAP throughout growth. Right-hand panels provide odds ratios along with 95% confidence intervals, where values in excess of 1 indicate higher mutability in exclusively early- versus exclusively late-bound sequence. Note that very few genomic regions are bound exclusively early (see Table S3). This applies to H-NS in particular so that odds ratio estimates are correspondingly noisy.

Two observations argue against these two models as satisfactory explanations. First, sequence bound throughout *all* stages of growth largely shows intermediate mutability (Figure S4). If stationary phase binding had been the only important variable, we would have expected mutability comparable to that in stationary phase. More importantly, regions bound exclusively early (during mid- and/or late-exponential but not later) or exclusively late (during transition to stationary and/or stationary phase but not earlier) show the most dramatic differences in mutability. Crucially, mutability in early-bound DNA commonly exceeds that of unbound sequence (Figure 3). If the only effect of NAP binding were to protect DNA from mutagenic processes, we would not expect mutation risk to ever exceed that of the unbound state, regardless of the timing of binding. Keeping in mind that the limited number of exclusively early-/late-bound sites precludes comprehensive confounder control, we suggest that protein occupancy during exponential growth actually impedes repair (or facilitates mutation, perhaps by interfering with polymerase processivity during replication).

### Evidence that NAP binding increases mutability during periods of active repair

**Figure 4:**
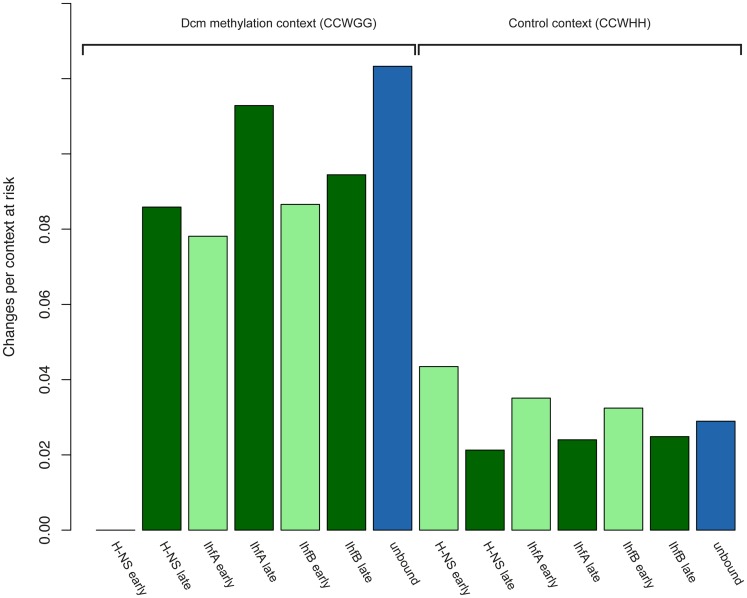
Mutability at 5-methylcytosine deamination hotspots. C∶G to T∶A mutability at the second cytosine in CCWGG motifs and CCWHH control motifs (expressed as changes per context at risk) for sequence bound by a specific NAP (either H-NS, IhfA, or IhfB) either exclusively early (light green) or exclusively late (dark green) or never (blue) bound by any of the NAPs considered (including Fis).

If NAP binding does, as suggested, interfere with repair and repair activity mainly occurred during exponential phase, that might explain why we observe elevated mutability during that stage of *E. coli* growth. Many key repair processes are indeed physically coupled to replication (3′-5′ exonucleolytic proof-reading of the DNA polymerase) or closely coupled in time (methyl-directed mismatch repair) and are down-regulated in stationary phase cells [35]. However, a limited number of repair enzymes are mainly expressed during stationary phase, including the alkylguaninetransferase Ada, which counteracts alkylation damage, the mismatch uracil glycosylase Mug, which reduces the incidence of C∶G to T∶A transitions and the very short patch VSP repair system (see below) [36]. Our hypothesis predicts that lesions specifically targeted by these enzymes should run counter to the overall trend and exhibit higher (not lower) mutability when bound during stationary phase. Is this the case? Testing this hypothesis is not trivial, the principal challenge being to identify mutations that have occurred (and should have been repaired by one of these enzymes) during stationary phase. Frequently, a variety of mutational processes, associated with diverse repair pathways, may have given rise to an observed nucleotide change. Pertinently, C∶G to T∶A changes can be caused by cytosine deamination to uracil, alkylation, or oxidation, result from deamination of 5-methylcytosine (5-meC) to thymine and even occur as a downstream consequence of UV-induced pyrimidine dimerization [37], [38]. Changes originating from 5-meC deamination are unique among these lesions because we can identify them based on the sequence context in which they occur. In *E. coli*, 5-meC is generated with high specificity and efficiency [39], [40] at the second cytosine of CCWGG motifs by the DNA cytosine methyltransferase Dcm. The T∶G mismatches that result from 5me-C deamination are repaired by the VSP repair pathway, which, importantly, is almost exclusively active during stationary phase [35], [41] and recognizes T∶G lesions outside the CCWGG context or U∶G mismatches created by cytosine deamination with much reduced efficiency [39], [42]. By implication, C to T changes that occurred in a CCWGG context are enriched for events that failed to be repaired during stationary phase whereas C to T changes outside that context more likely represent failures by other repair pathways such as methyl-directed mismatch or base excision repair, which are most active during exponential growth [35], [43]. We therefore compared C to T changes that occurred in a CCWGG context with changes in a control context (CCWHH) chosen to preserve local nucleotide neighbourhood. Focussing on exclusively late versus exclusively early bound sequences, which afford the greatest discriminatory power, we find that the control contexts exhibit the mutability behaviour observed previously (refractory effects of NAP occupancy during exponential phase, Figure 4). However, as predicted, changes that occurred in the CCWGG context show the opposite pattern, i.e. higher mutability for sequence bound during stationary phase. Observing higher mutability in late-bound regions inside the CCWGG context but lower mutability outside of it is unlikely to occur by chance (permutation test: P = 0.013, see Methods). Once again, it is important to highlight that sites exclusively bound early or late are rare so that we cannot control systematically for potential confounders. However, on current evidence, this finding supports the hypothesis that NAP binding enhances mutability by interfering with repair pathways in a lesion- and growth phase-specific manner.

## Discussion

It has been known for some time that both the rate [44] and spectrum [45] of mutations vary with growth stage in laboratory populations of *E. coli* and other bacteria, consistent with substantial physiological variability in the production of endogenous mutagens, differential activity of mutagenic processes such as replication or recombination, and variable expression of DNA repair enzymes. We argue that taking this physiological variability into account is crucial to understand the impact of DNA-binding proteins on sequence evolution in natural populations. This is because binding has pleiotropic effects on mutability, is capable of both protecting DNA from mutagens and interfering with lesion repair, so reducing or enhancing mutability in a dynamic fashion that depends on the physiological state of the cell.

The analysis presented above suggests that the net effect of NAP binding on mutability is relatively weak and NAP binding is a comparatively poor predictor of within-genome differences in mutability. However, one should not jump to the conclusion that NAPs are largely irrelevant determinants of mutation dynamics on a mechanistic level. As indicated by the comparison between sites bound exclusively during exponential or stationary phase (Figure 3), and consistent with biochemical evidence [16], NAPs can exert countervailing effects on DNA mutability, elevating mutation risk under one condition but reducing it in another. We suggest that, over the bacteria's life cycle, and evolutionary time, these forces partially counteract each other, leading to a relatively small net difference between NAP-bound and NAP-free sequence. Note also, that our analysis is very conservative. Some of the confounders might in fact be mechanistically linked to NAP binding. For example, it has been suggested that Fis binding changes repair dynamics at the tyrT locus through altering transcription patterns [16]. We would attribute such an effect to transcription rather than NAP binding therefore understating the significance of NAP binding.

Our analysis also suggests that only a subset of mutation processes are affected by NAP binding. Given the plethora of lesion processes, some of which may be more, some less affected by DNA curvature and protein binding, it is not surprising that not all mutation types behave in a similar fashion. However, it is interesting to ask why we specifically observe an effect for C to G, G to C, T to C, and C to T changes. C∶G to G∶C transversions are rare and there is considerable uncertainty as to which lesions predominantly give rise to these mutations in the wild [46]. Guanines damaged by oxidative and alkylation processes can lead to C∶G to G∶C mutations [38], [47], but it is hard to see why NAP binding should preferentially reduce the incidence of oxidative lesions leading to C∶G to G∶C mutations but not affect mutability for C∶G to A∶T transversions, which are frequently derived from oxidatively damaged bases. Perhaps differences in lesion repair provide a more likely link to NAP binding. C∶C mismatches, which – if unrepaired – would yield C∶G to G∶C transversions, exhibit intrinsically high mobility compared to other mispaired bases [48]. It is conceivable that these mismatches are stabilized in the context of NAP binding, facilitating their detection and repair. However, this is speculative and we currently have no convincing model why NAP binding specifically affects C∶G to G∶C mutability.

Reduced rates of C to T transitions might be owing to an effect of NAP binding on cytosine deamination dynamics, analogous to what has been proposed for nucleosomes in eukaryotes [6]. Comparing base-specific mutability in *E. coli* with base-specific substitution rates at 4-fold synonymous sites in yeast reveals that C∶G to T∶A transitions, the most common type of nucleotide replacement, are less likely to occur in a protein-bound context in both taxa (Figure S5). Although it remains unclear to what extent nucleosome-related substitution dynamics in yeast [20], [23] and other eukaryotes [21] are modulated by selection, recent data [6] suggest that elevated C∶G to T∶A rates in nucleosomal DNA principally reflect mutational input rather than selection. That does not automatically imply, of course, that protective effects in *E. coli* and eukaryotes are mediated by the same mechanism. However, it is tempting to speculate that both NAPs and nucleosomes reduce C∶G to T∶A mutability by limiting the amount of time the bound DNA spends in a vulnerable single-stranded state [6]. Such a model, where protection is not contingent on a specific protein-DNA binding conformation, is attractive because we observe consistent trends across proteins that differ substantially in how they bind to DNA and affect its topology [9]. Beyond C∶G to T∶A changes, there seems to be limited agreement in mutability trends between yeast and *E. coli* (Figure S5). However, this comparison is preliminary and a comprehensive assessment of convergent mutability will require a thorough, confounder-controlled analysis of mutability patterns in yeast.

## Methods

### Multiple genome alignment

We extracted all complete *E. coli* and Shigella genomes available in GenBank. Excluding a number of genomes known to be of inferior quality [49], we arrived at a final set of 54 *E. coli*/Shigella genomes (Table S1). These genomes were aligned along with the genome of *E. fergusonii* using progressiveMauve [50]. We then only considered alignment blocks (locally collinear blocks in Mauve terminology) present across all 55 genomes. Further, we only considered blocks that did not overlap non-unique regions of the *E. coli* K-12 MG1655 genome. This is because binding calls from the Chip-Seq experiments only considered reads that uniquely mapped to the MG1655 genome so that – if we included non-unique regions – we would consider them unbound although they might in fact be bound. Non-unique portions of the *E. coli* genome were defined by subjecting the MG1655 genome to *in silico* digestion into overlapping windows of 300 nucleotides (off-set by 10 nt). These pseudo-reads were mapped against the MG1655 genome using GEM (http://sourceforge.net/apps/mediawiki/gemlibrary/index.php?title=The_GEM_library) allowing up to 2 mismatches. In cases where pseudo-reads mapped to more than one genomic locale, these locales were excluded from downstream analysis. This procedure eliminates only a small fraction of the MG1655 genome (∼2%).

Polymorphic sites were extracted using Mauve and filtered to obtain a set of sites where none of the species contained a gap and polymorphic status was not caused by a difference in *E. fergusonii*, i.e. we required a polymorphic site amongst the *E. coli*/Shigella genomes.

### Phylogenetic reconstruction

Locally collinear blocks were concatenated, homomorphic positions removed and a phylogenetic tree constructed using RAxML 7.2.8 [51], [52] with 100 rapid bootstraps followed by thorough maximum likelihood search. The topology of the best tree (Figure S6) closely resembles the topologies of previously reported multi-strain *E. coli* trees [33], [53].

### Reconstruction of nucleotide changes

Considering *E. coli* genomes in a phylogenetic context, we reconstructed the history of nucleotide changes using the baseml algorithm in PAML [54]. The tree obtained above was used as a guide tree. We then applied the following filters to obtain a set of high confidence changes: a) only a single event had to be evoked to explain the distribution of states through the phylogeny, b) the reconstructed ancestral state had a posterior probability > = 0.9, c) the change is not dependent on topology downstream of poorly supported (bootstrap support <98%) nodes (see Figure S6). These filters, along with requiring coverage across all 55 genomes, are designed to reduce the impact of false inferences caused by inter-strain recombination/horizontal gene transfer as well as sequencing and alignment errors.

### NAP binding

Genome-wide binding profiles for four nucleoid-associated proteins (H-NS, Fis, IhfA, and IhfB) were obtained from two publications (REFs. 29 and 30). The authors determined binding profiles at four points during colony growth: during mid-exponential (OD = 0.5, Prieto, pers. comm.), late exponential (OD = 0.8–0.9), transition to stationary (OD = 1.8–2), and stationary phase (after 24 hours).

### Classifier and statistical analysis

In order to establish whether NAP occupancy has an effect on mutability that can be separated from potential confounding factors we took the following approach: We trained Random Forest (RF) classifiers [55] to separate instances where a certain change at a 4-fold synonymous site (e.g. C to T) had occurred along the *E. coli* phylogeny from instances where the nucleotide at risk (C in our example) had not experienced a change. The (very large) class of unchanged nucleotides was randomly sub-sampled to obtain a sample exactly five times larger than the (smaller) changed-nucleotide class, which was kept intact. All 12 possible nucleotide changes were classified independently. The settings of the RF classifier were as follows: forest size was set to a very large value (10,000 trees) to avoid limiting predictive accuracy; the maximum tree depth was slightly decreased from the default setting (unlimited depth) to 10, for reasons of computational efficiency. While RF is considered robust to choice of parameters, one particular parameter may influence its predictive ability somewhat, namely the number of features considered at each node [55], here called *K*. Therefore we optimized *K* to maximize the average cross-validation accuracy of the RF models across the 12 nucleotide changes, yielding *K* = 2 as the optimum (Figure S7). A higher (5∶1) RF weight was put on the minority class (mutated nucleotides) than on the majority class (unchanged nucleotides) to counter the imbalance in the number of sampled nucleotides. Other RF parameters were left at the default values. 2-fold synonymous sites were included when analyzing transitions. The classifier was provided with a number of features potentially predictive of mutability such as the location of the corresponding gene relative to the origin of replication and its expression level at a certain stage of growth (see Table S2 for a full list of features). We recoded growth phase-specific NAP binding into four categories, bound throughout the growth cycle (always), bound during mid-exponential or mid- and late exponential phase (early), bound during stationary or stationary and transition to stationary phase (late) or not bound at any time point (never). While this approach discards more complex but potentially genuine binding behaviour, it captures the majority of binding profiles observed in the data (see Table S3) and reduces the number of binding categories, hence increasing power.

We then assessed classifier performance in the presence of all features, including NAP binding, as the area under the curve (AUC). We then repeated the classification process 50 times, each time randomly shuffling NAP binding status across residues. The approximately normal distribution of AUC values from these randomized runs was then compared to the AUC derived from observed data by finding a Z-score to establish whether considering NAP binding improves classifier performance beyond confounding factors. A one-tailed P value was then derived from the Z-score. A one-tailed test is appropriate here because the only relevant outcome of the test is a decrease in the AUC score upon randomization. Note that it does not matter whether NAP binding is positively or negatively correlated with mutation risk; the non-randomized AUC score should always be higher. With 10% false positives expected at the P<0.1 threshold, corresponding to 0.6 false positive results from the 6 mutation types (3 couples) that were found associated with protein binding in our univariate odds ratio test (Figure 1), and 4 mutation types testing positive below the P<0.1 threshold in the RF randomization test, we estimate a false discovery rate of FDR = 0.6/4 = 15%.

To establish whether the pattern depicted in Figure 4 (higher mutability across NAPs for late-bound residues in CCWGG contexts but lower mutability for late-bound residues in control contexts) is likely to arise by chance, we adopted the following strategy: we preserved the total number of mutations that occurred within each NAP+context combination (e.g. H-NS+control context) but redistributed mutations randomly to the late- or early-bound category according to the number of contexts in each category. Repeating this procedure 100,000 times for all six NAP+context combinations, we asked how likely we are to observe all NAP+context pairs to conform to the observed pattern.

All classifier analyses were carried out within the Weka machine learning suite [56] and a customized version of the FastRandomForest classifier (http://fast-random-forest.googlecode.com/). All statistical analyses were conducted in R [57].

## Supporting Information

Figure S1 **The frequency spectrum of nucleotide changes at 4-fold synonymous sites resembles the frequency spectrum observed in mutation accumulation experiments.** The left panel corresponds to the central panel of Figure 1 in the main text, but pooling changes that occurred at bound and unbound sites. The right panel gives the frequencies of different mutation types (per at-risk nucleotide), as determined across the entire MG1655 genome in a recent mutation accumulation experiment [34]. A total of 233 mutations were observed in this experiment.
(EPS)

Figure S2 **The effect of NAP binding status on classifier performance.** Z scores indicate the difference between classifier performance when *bona fide* NAP binding is considered compared to 50 runs with randomized binding status. Positive Z scores reflect increased performance. Mutation classes that show a significant drop-off in classifier performance when binding status is ignored are marked with * (P<0.1) or ** (P<0.05). Mutation couples that exhibit significantly different odds ratios for bound versus unbound sequence (see Figure 1 in the main text) are highlighted in red (one-tailed test, see Materials and Methods). Expectedly, none of the mutation couples that did not show significant odds ratios in the univariate analysis in Figure 1 (here in black) appear affected by NAP binding.
(EPS)

Figure S3 **Overlap in binding regions across growth phases.** The bars show the fraction of sequence bound during stationary phase that is also occupied by the same protein (IhfA, IhfB, or H-NS) during transition to stationary, late exponential and mid-exponential phase.
(EPS)

Figure S4 **DNA bound by NAPs throughout the growth cycle exhibits intermediate mutability.** This figure corresponds to Figure 2 in the main text. Grey bars represent regions bound by a given NAP during all four growth stages sampled.
(EPS)

Figure S5 **Mutability at 4-fold synonymous sites as a function of NAP (nucleosome) occupancy in** ***E. coli*** **(** ***S. cerevisiae*** **).** The left panel shows mutability (changes per nucleotide at risk) as a function of NAP occupancy for all possible transitions and transversions. White: sequence bound by one of the four NAPs. Sequence is considered bound when at least one of the NAPs binds during at least one of the growth phases assayed (see main text). Blue: sequence not bound by any of the four NAPs throughout growth. On the right, mutability as a function of nucleosome occupancy in *S. cerevisiae*. Base-specific rates correspond to rates reported for the highest (white) and lowest (blue) nucleosome occupancy bin in supplementary figure 7 of Chen et al. [6]. To what extent differences in A∶T to G∶C mutability between *E. coli* and yeast genuinely reflect differences in protein occupancy or alternative confounders between the two taxa remains to be established.
(EPS)

Figure S6 **Maximum likelihood tree of 54** ***E. coli*** **/Shigella genomes.** *E. fergusonii* was used to root the tree (see Methods for details of tree reconstruction).
(EPS)

Figure S7 **Finding the optimal value of the Random Forest parameter** ***K*** **, the number of features considered for each split.** *K* can vary from 1 to the total number of features (12) in increments of 1. The optimum *K* is defined as the one yielding the highest average normalized AUC score across the 12 mutation datasets. The AUC score is obtained from the Random Forest's out-of-bag cross-validation procedure, and normalized to range from 0 to 1 within each mutation dataset separately; then, the average is computed, which was found to be highest for K = 2. The range of original, non-normalized AUC scores for each dataset is given in parentheses.
(PDF)

Table S1 **Escherichia strains and their chromosomal accessions.**
(PDF)

Table S2 **Potential predictors of mutability considered in the classifier analysis.**
(PDF)

Table S3 **Encoding growth phase-specific binding.**
(PDF)
