# Supplementary figures and images for: Nucleoid-Associated Proteins Affect Mutation Dynamics in E. coli in a Growth Phase-Specific Manner
Source: PLoS Comput Biol. 2012 Dec 20;8(12):e1002846. doi: 10.1371/journal.pcbi.1002846 (PMC3527292; doi:10.1371/journal.pcbi.1002846)

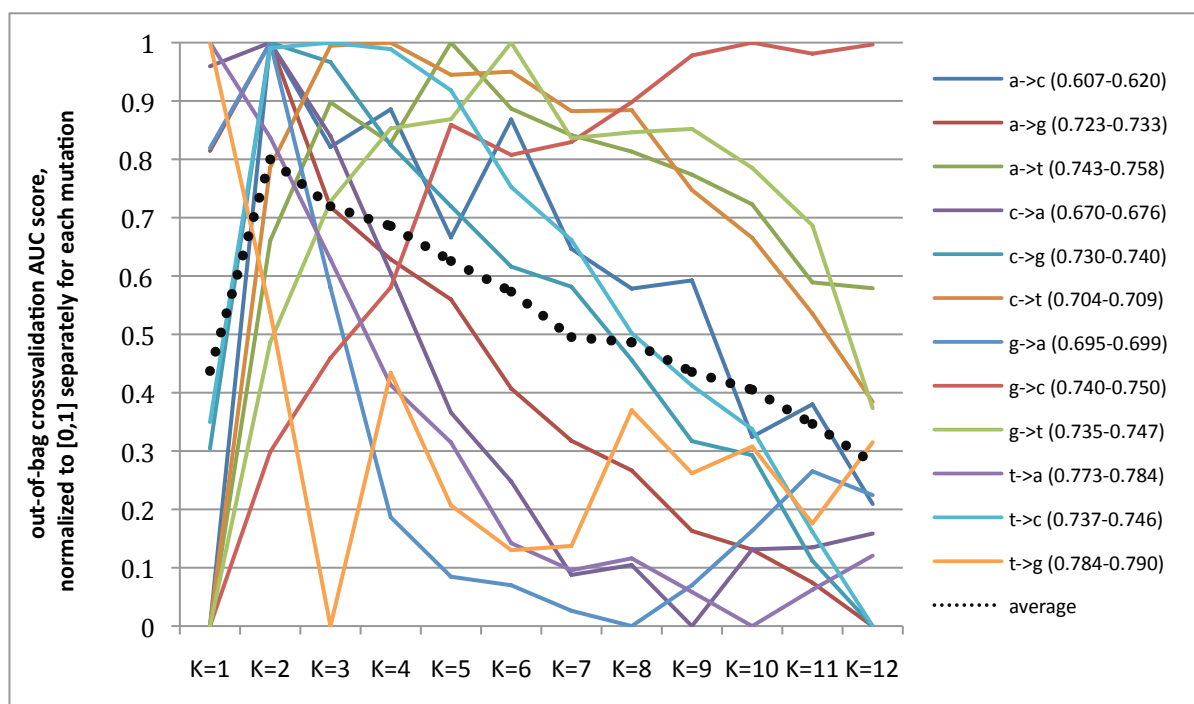

Supplement: Figure S7 — Finding the optimal value of the Random Forest parameter K , the number of features considered for each split. K can vary from 1 to the total number of features (12) in increments of 1. The optimum K is defined as the one yielding the highest average normalized AUC score across the 12 mutation datasets. The AUC score is obtained from the Random Forest's out-of-bag cross-validation procedure, and normalized to range from 0 to 1 within each mutation dataset separately; then, the average is computed, which was found to be highest for K = 2. The range of original, non-normalized AUC scores for each dataset is given in parentheses. (PDF) [file pcbi.1002846.s007.pdf]
