# Supplementary material for: Nucleoid-Associated Proteins Affect Mutation Dynamics in E. coli in a Growth Phase-Specific Manner
Source: PLoS Comput Biol. 2012 Dec 20;8(12):e1002846. doi: 10.1371/journal.pcbi.1002846 (PMC3527292; doi:10.1371/journal.pcbi.1002846)
Supplement: Table S1 — Escherichia strains and their chromosomal accessions. (PDF) [file pcbi.1002846.s008.pdf]

Table S1. Escherichia strains and their chromosomal accessions

Strain name	RefSeq/GenBank accession
Escherichia fergusonii ATCC 35469	NC_011740
Escherichia coli str. K-12 substr. MG1655	NC_000913.2
Escherichia coli BL21(DE3)	AM946981.2
Escherichia coli SE15	AP009378.1
Escherichia coli DH1	AP012030.1
Escherichia coli BL21(DE3)	CP001509.3
Escherichia coli DH1	CP001637.1
Escherichia coli ABU 83972	CP001671.1
Escherichia coli O83:H1 str. NRG 857C	CP001855.1
Escherichia coli IHE3034	CP001969.1
Escherichia coli UM146	CP002167.1
Escherichia coli W	CP002185.1
Escherichia coli str. 'clone D i2'	CP002211.1
Escherichia coli str. 'clone D i14'	CP002212.1
Escherichia coli UMNK88	CP002729.1
Escherichia coli NA114	CP002797.2
Escherichia coli O7:K1 str. CE10	CP003034.1
Escherichia coli O55:H7 str. RM12579	CP003109.1
Escherichia coli LF82	CU651637.1
Escherichia coli 042	FN554766.1
Escherichia coli ETEC H10407	FN649414.1
Escherichia coli UTI89	NC_007946

Escherichia coli 536	NC_008253
Escherichia coli APEC O1	NC_008563
Escherichia coli HS	NC_009800
Escherichia coli E24377A	NC_009801
Escherichia coli ATCC 8739	NC_010468
Escherichia coli str. K-12 substr. DH10B	NC_010473
Escherichia coli SMS-3-5	NC_010498
Escherichia coli O157:H7 str. EC4115	NC_011353
Escherichia coli SE11	NC_011415
Escherichia coli O127:H6 str. E2348/69	NC_011601
Escherichia coli IAI1	NC_011741
Escherichia coli S88	NC_011742
Escherichia coli ED1a	NC_011745
Escherichia coli 55989	NC_011748
Escherichia coli IAI39	NC_011750
Escherichia coli UMN026	NC_011751
Escherichia coli BW2952	NC_012759
Escherichia coli 'BL21-Gold(DE3)pLysS AG'	NC_012947
Escherichia coli B str. REL606	NC_012967
Escherichia coli O157:H7 str. TW14359	NC_013008
Escherichia coli O103:H2 str. 12009	NC_013353
Escherichia coli O26:H11 str. 11368	NC_013361
Escherichia coli O111:H- str. 11128	NC_013364
Escherichia coli O55:H7 str. CB9615	NC_013941

Escherichia coli KO11FL	NC_016902
Shigella sonnei Ss046	NC_007384
Shigella sonnei 53G	NC_016822
Shigella dysenteriae Sd197	NC_007606
Shigella boydii Sb227	NC_007613
Shigella boydii CDC 3083-94	NC_010658
Shigella flexneri 5 str. 8401	NC_008258
Shigella flexneri 2a str. 301	NC_004337
Shigella flexneri 2002017	CP001383.1
