# Supplementary material for: Nucleoid-Associated Proteins Affect Mutation Dynamics in E. coli in a Growth Phase-Specific Manner
Source: PLoS Comput Biol. 2012 Dec 20;8(12):e1002846. doi: 10.1371/journal.pcbi.1002846 (PMC3527292; doi:10.1371/journal.pcbi.1002846)
Supplement: Table S2 — Potential predictors of mutability considered in the classifier analysis. (PDF) [file pcbi.1002846.s009.pdf]

Table S2 – Potential predictors of mutability considered in the classifier analysis

Feature	Values
NAP binding	Bound by any NAP early, late, always or never*
Strand	Strand on which the corresponding gene is located
Distance to the origin	Shortest distance (clock- or counter-clockwise) to the origin
CDS length	Length of the coding sequence in nucleotides
Upstream nucleotide	Identity of the upstream nucleotide (in relation to the orientation of the reading frame)<br>• one of A, C, G, or T
Downstream nucleotide	Identity of the downstream nucleotide (in relation to the orientation of the reading frame)<br>• one of A, C, G, or T
Distance from CDS start	The distance from the start of the coding sequence in nucleotides

Distance from CDS end	The distance from the end of the coding sequence in nucleotides
Expression level prior to stationary phase	Mean expression level across replicates in mid- and late exponential and transition to stationary phase as determined by [31,32]
Expression level during stationary phase	Mean expression level across replicates during stationary phase as determined by [31,32]
Regional GC content	GC content in a window of ±500 nucleotides around the focal site
Local GC content	GC content in a window of ±50 nucleotides around the focal site

*see Table S3 for precise encoding
