# Supplementary material for: Nucleoid-Associated Proteins Affect Mutation Dynamics in E. coli in a Growth Phase-Specific Manner
Source: PLoS Comput Biol. 2012 Dec 20;8(12):e1002846. doi: 10.1371/journal.pcbi.1002846 (PMC3527292; doi:10.1371/journal.pcbi.1002846)
Supplement: Table S3 — Encoding growth phase-specific binding. (PDF) [file pcbi.1002846.s010.pdf]

Table S3. Encoding growth phase-specific binding

Bound* during mid-exponential phase	Bound during late exponential phase	Bound during transition to stationary phase	Bound during stationary phase	Category	Number of changes/synonymous sites in category†
✓	✓	✓	✓	Always	1,155/74,612
×	×	×	×	Never	7,244/543,086
×	×	✓	✓	Late	157/13,565
×	×	×	✓	Late	396/32,478
✓	×	×	×	Early	351/19,734
✓	✓	×	×	Early	652/37,562
✓	✓	✓	×	Discarded	52/3,703
✓	×	✓	✓	Discarded	16/1,308
✓	✓	×	✓	Discarded	57/5,254
✓	×	×	✓	Discarded	28/1,250
✓	×	✓	×	Discarded	6/259
×	×	✓	×	Discarded	25/1,998
×	✓	×	×	Discarded	254/16,598
×	✓	✓	✓	Discarded	223/23,859
×	✓	✓	×	Discarded	19/1,183
×	✓	×	✓	Discarded	40/3,712

*by any of the four NAPs considered
†includes 2-fold synonymous sites for transitions
